# Transcriptomic analysis reveals the molecular mechanisms of rumen wall morphological and functional development induced by different solid diet introduction in a lamb model

**DOI:** 10.1186/s40104-021-00556-4

**Published:** 2021-03-10

**Authors:** Daming Sun, Yuyang Yin, Changzheng Guo, Lixiang Liu, Shengyong Mao, Weiyun Zhu, Junhua Liu

**Affiliations:** 1 Jiangsu Key Laboratory of Gastrointestinal Nutrition and Animal Health, Laboratory of Gastrointestinal Microbiology, College of Animal Science and Technology, Nanjing Agricultural University, Nanjing, 210095 Jiangsu Province China; 2 National Center for International Research on Animal Gut Nutrition, Nanjing Agricultural University, Nanjing, 210095 China; 3 National Experimental Teaching Demonstration Center of Animal Science, Nanjing Agricultural University, Nanjing, 210095 China; 4 Huzhou Academy of Agricultural Sciences, Huzhou, 313000 China

**Keywords:** Concentrate, Hay, Lamb, Rumen development, Transcriptome

## Abstract

**Background:**

This study aimed to elucidate the molecular mechanisms of solid diet introduction initiating the cellular growth and maturation of rumen tissues and characterize the shared and unique biological processes upon different solid diet regimes.

**Methods:**

Twenty-four Hu lambs were randomly allocated to three groups fed following diets: goat milk powder only (M, *n* = 8), goat milk powder + alfalfa hay (MH, *n* = 8), and goat milk powder + concentrate starter (MC, *n* = 8). At 42 days of age, the lambs were slaughtered. Ruminal fluid sample was collected for analysis of concentration of volatile fatty acid (VFA) and microbial crude protein (MCP). The sample of the rumen wall from the ventral sac was collected for analysis of rumen papilla morphology and transcriptomics.

**Results:**

Compared with the M group, MH and MC group had a higher concentration of VFA, MCP, rumen weight, and rumen papilla area. The transcriptomic results of rumen wall showed that there were 312 shared differentially expressed genes (DEGs) between in “MH vs. M” and “MC vs. M”, and 232 or 796 unique DEGs observed in “MH vs. M” or “MC vs. M”, respectively. The shared DEGs were most enriched in VFA absorption and metabolism, such as peroxisome proliferator-activated receptor (PPAR) signaling pathway, butanoate metabolism, and synthesis and degradation of ketone bodies. Additionally, a weighted gene co-expression network analysis identified M16 (2,052 genes) and M18 (579 genes) modules were positively correlated with VFA and rumen wall morphology. The M16 module was mainly related to metabolism pathway, while the M18 module was mainly associated with signaling transport. Moreover, hay specifically depressed expression of genes involved in cytokine production, immune response, and immunocyte activation, and concentrate starter mainly altered nutrient transport and metabolism, especially ion transport, amino acid, and fatty acid metabolism.

**Conclusions:**

The energy production during VFA metabolism may drive the rumen wall development directly. The hay introduction facilitated establishment of immune function, while the concentrate starter enhanced nutrient transport and metabolism, which are important biological processes required for rumen development.

**Supplementary Information:**

The online version contains supplementary material available at 10.1186/s40104-021-00556-4.

## Background

Rumen, as the specific digestive and metabolism organ in ruminants, is responsible for feed degradation, nutrient absorption and metabolism, and immune response, where microbial fermentation produces volatile fatty acids (VFAs), microbial proteins, vitamins, and other nutrients [1, 2]. Volatile fatty acids in the rumen contribute up to 70% energy requirements of the host [3], and microbial proteins account for up to 90% of amino acids utilized for host protein synthesis [4]. Therefore, a well-developed rumen is critical for ruminant health and performance. Rumen development process consists of microbial colonization, functional achievement, as well as anatomic development [5]. Microbial colonization performs efficient digestion of feed components, thereby providing nutrients for the physiological requirements of the animal [5]. Rumen wall mainly functions as efficient absorption and metabolism of VFA, maintains rumen homeostasis, and provides energy to host [6]. In addition, rumen wall has an important role in immune and barrier functions, thus, its integrity is essential to ensure the health of ruminants [1]. The transition phase (pre-ruminant) of the young ruminant is the sensitive window time to manipulate rumen wall development. Thus, improving rumen wall morphology and physiological function development by nutritional strategies during this early period has great significance for ruminant life-time health and production.

In newborn ruminants, suckling liquid feed (milk or milk replacer) mainly flows into the abomasum through the esophageal groove, resulting in less fermentation substrate stimulating rumen development [7, 8]. Hay and concentrate starter are the main diet components for improving rumen wall morphological development in calves and lambs’ production. Previous studies showed that concentrate starter introduction had larger emptied rumen weight and papilla surface than hay in pre-weaned calf, and hay introduction especially increased the volume of the rumen [9–11]. However, the molecular mechanism of rumen wall morphological and functional development is not well understood. At the molecular level, a previous study indicated that, compared with concentrate starter, hay plus concentrate starter introduction enhanced expression of genes related to VFA absorption in the ruminal epithelia of calves [12]. Our recent study showed that, compared with hay introduction, hay plus concentrate starter introduction increased expression of genes associated with cell proliferation, while decreased the expression of genes related to VFA absorption [11]. This indicated that hay and concentrate may have their own unique effects on the molecular processes of rumen morphological and functional development in pre-weaning ruminants. By transcriptomics technology, emerging evidence has shown that hay feeding activated gene pathways participating in energy production [13], and hay plus concentrate starter introduction enhanced amino acid and fatty acid metabolic processes in the rumen tissue of lambs during pre-weaning period [14]. Compared to calves fed with only milk replacer before 42 days of age, calves with concentrate introduction from 42 to 56 days of age activated molecular pathways primarily related to the cell cycle in the rumen wall [13]. Also, hay plus concentrate starter introduction activated gene pathways participating in growth and development in the rumen epithelium of lambs [15]. These studies indicated that hay or concentrate starter introduction may affect specific molecular and biological processes in the morphological and functional development of rumen wall. However, these studies mixed the age and diet factors, so that it is not confirmed that these changes are mainly derived from hay or concentrate introduction. Furthermore, it is unclear how hay or concentrate starter introduction drives differently morphological and functional development in the rumen wall of young ruminants.

This study aimed to investigate the molecular mechanisms of rumen wall morphological and functional development induced by different solid diet introduction, and to characterize the specific molecular and biological processes upon introduction of hay or concentrate.

## Methods

### Animal experiment design

The experimental design and procedures were approved by the Animal Care and Use Committee of Nanjing Agricultural University. Twenty-four 12-day-old Hu lambs (12 males and 12 females) were separated from their dams and fed mixed goat milk powder (water: goat milk powder = 10:1) in individual pens. At 15 days of age, the lambs were randomly assigned by sex to three groups fed following diets: goat milk powder only (M, *n* = 8), goat milk powder + alfalfa hay (MH, *n* = 8), and goat milk powder + concentrate starter (MC, *n* = 8). The lambs in the M group had access to mixed goat milk powder (23.85% crud protein, 25.30% fat, and 36.30% lactose) for 1 h at each feeding time. The feeding amount of milk powder per lamb in MH and MC group (600 mL/d) was provided according to previous research, which was equal to 10% of their initial average body weight (BW, about 6 kg) [10, 16] and could freely get access to alfalfa hay or concentrate starter, respectively. All the lambs were fed mixed goat milk four times a day (07:00, 12:00, 17:00, and 22:00) and had ad libitum access to water. Lambs in MH and MC group were fed with 200 g/d of solid diet during the first 2 weeks, followed by 500 g/d during the next 2 weeks. The solid diet was offered in equal amounts at 08:00 and 17:00 daily. The dry matter intake of diet was recorded every day. Nutritional levels of alfalfa and concentrate starter are shown in Table 1.

**Table 1:** Ingredient and chemical composition of the diets for alfalfa hay group (MH) and concentrate starter group (MC) lambs (dry matter basis)

Item	MH	MC
Ingredients, % DM		
Alfalfa hay	98.50	0.00
Corn	0.00	56.00
Soybean meal	0.00	31.00
Wheat	0.00	4.00
Whey powder	0.00	5.00
limestone meal powder	0.00	1.00
Calcium monophosphate	0.00	1.50
Nacl	0.50	0.50
Premix^a^	1.00	1.00
Nutrient composition		
Metabolic energy, MJ/kg DM^b^	8.03	12.67
Crude protein, % DM	18.76	19.62
Ether extract, % DM	2.26	3.68
Crude fiber, % DM	22.29	3.35
Crude ash, % DM	7.46	3.16
NDF, % DM	36.45	10.23
ADF, % DM	30.54	5.11
Starch, % DM	1.00	35.64
Ca, % DM	1.38	1.04
P, % DM	0.50	0.50

^a^Contained 102 g/kg of Zn, 47 g/kg of Mn, 26 g/kg of Cu, 1140 mg/kg of I, 500 mg/kg of Se, 340 mg/kg of Co, 17,167,380 IU/kg of vitamin A, 858,370 IU/kg of vitamin D, and 23,605 IU/kg of vitamin E

^b^Calculated based on Ministry of Agriculture of China recommendations (MOA, 2004). DM, dry matter

### Sample collection

At 42 days of age, the jugular vein blood of lambs was collected using a blood collection tube containing 40 KIU Na-heparin/mL blood at 2 to 4 h after the last diet feeding. Plasma was harvested by centrifuging the blood samples at 3,000 r/min at 4 °C for 10 min and stored at − 20 °C. Then, lambs were stunned by electric shock and killed by exsanguinations. Immediately after slaughter, rumen was opened by an incision along the dorsal curvature. All rumen content was mixed for determining the pH value and then was strained through four layers of cheesecloth for sampling ruminal fluid. The ruminal fluid samples were stored at − 20 °C until analysis for the concentrations of VFAs, ammonia nitrogen (NH_3_-N), and microbial crude protein (MCP). After recording the weight and volume of the rumen, rumen wall (3 cm × 3 cm) sample from the ventral sac was collected for analysis of rumen papilla morphology, histomorphometry microscopy, and transcriptomics as described previously [11, 14, 15].

### Physiological parameter measurements

Ruminal pH and VFA concentration were detected by portable pH meter (HI 9024C; HANNA Instruments, Woonsocket, RI, USA) and capillary column gas chromatography (GC-14B; Shimadzu, Tokyo, Japan), respectively. The concentrations of NH_3_-N [17] and MCP [18] were analyzed according to previous studies. Rumen volume [19], morphometric and histomorphometric microscopy of the rumen wall [10, 20] were examined as previously described. The plasma concentrations of insulin-like growth factor 1, β-hydroxybutyric acid (BHBA), insulin, and glucose were analyzed by the commercial kits (Jiancheng Bioengineering Institute, Nanjing) following the manufacturer’s protocols.

### RNA extraction and sequencing

Four rumen tissue samples per group were randomly selected to conduct transcriptome analysis. Total RNA of rumen tissues sample was isolated by TRIzol (Invitrogen Life Technologies, Carlsbad, CA, USA), and RNA concentrations and quality were examined by NanoDrop (NanoDrop Technologies, Wilmington, DE, USA). A total amount of 1.5 μg RNA per sample was used for cDNA library construction [21]. The ribosomal RNA was removed from total RNA using the Ribo-Zero™ Kit (Epicentre, Madison, WI, USA). Then, sequencing libraries were generated using NEBNext® Ultra™ Directional RNA Library Prep Kit for Illumina (NEB, USA) following manufacturer’s recommendations, and index codes were added to attribute sequences to each sample. The clustering of the index-coded samples was performed on the acBot Cluster Generation System using TruSeq PE Cluster Kit V3-cBot-HS (Illumina) according to the manufacturer’s instructions. After cluster generation, the library preparations were sequenced on an Illumina Hiseq platform and paired-end reads were generated.

### Transcriptome analysis

An in-house perl script was used to remove low-quality reads. HISAT2 was used to align the remaining reads to the host [22]. The software StringTie (version 1.3.1) was used to map reads to estimate the expression of each gene transcript [22, 23]. Gene expression levels were estimated by fragments per kilobase of transcript per million fragments mapped (FPKM). Differential expression analyses in the MH and MC groups were performed using the DESeq R package (1.10.1). To count the differentially expressed genes (DEGs), an adjusted *P* < 0.05 and an absolute value of [log_2_ (fold change)] > 1 were set as the filter criteria for significantly differential expression. The gene ontology (GO) enrichment analysis of DEGs was carried out by DAVID (version 6.8) [24]. KOBAS (version 3.0) was used to test the statistical enrichment of DEGs in the Kyoto Encyclopedia of Genes and Genomes (KEGG) pathways [25].

### Weighted gene co-expression network analysis

Weighted gene co-expression network analysis (WGCNA) was performed to understand the correlation of host transcriptome with the rumen fermentation parameters and development indexes. All expressed genes (16,329, FPKM > 0.1 in at least 1 sample) in rumen tissue samples collected from all lambs were used in WGCNA analysis (R studio v1.3.1335). After applying the soft thresholding power, a signed network was constructed using the Pearson correlation. Subsequently, a Pearson correlation analysis of genes expression with the rumen fermentation parameters and development indexes of each module (clusters of highly interconnected genes) was performed. Module detection (blockwise modules in WGCNA) functions were performed with the following parameters: max module size was set to 12,000 genes and minimum module size set to 150 genes, and a reassign threshold of 0.25.

### Statistical analysis

The results of animal performance, ruminal fermentation, blood parameter, and rumen development parameters were analyzed using the one-way ANOVA model in SPSS software (SPSS version 25.0, SPSS, Inc.), with different diet as the main factor. The differences among means were detected by the Turkey’s multiple range test. The results presented as means with standard error of the means (SEM). A value of *P* < 0.05 was regarded as statistically significant. The DEGs based on transcriptome analysis were analyzed using the Kruskal-Wallis in SPSS software. The *P* values were adjusted using the Benjamini-Hochberg method. An adjusted *P* < 0.05 and an absolute value of [log_2_ (fold change)] > 1 were set as the filter criteria for significantly differential expression. Venn analysis was conducted using Venny 2.1 (https://bioinfogp.cnb.csic.es/tools/venny/ index.html).

## Results

### Animal performance

As shown in Supplemental Fig. 1, BW (*P* = 0.157) of lambs had no significant difference among the three groups at the end of experiment. Compared with MC lambs, the M lambs had lower average daily feed intake (based on dry matter, *P* = 0.032), but there was no significant difference between M and MH lambs during the experiment. The MH lambs had lower average daily gain (ADG) (*P* = 0.019) than that in MC lambs, but there was no significant difference between M and MH lambs during the experiment.

### Ruminal fermentation and blood parameter

As shown in Table 2, the concentrations of total VFA (*P* < 0.001), acetate (*P* < 0.001), propionate (*P* < 0.001), butyrate (*P* < 0.001), valerate (*P* < 0.001), and MCP (*P* < 0.001) were the highest in the rumen in MC lambs, followed by MH lambs, and then M lambs. The concentrations of isobutyrate (*P* < 0.001) and isovalerate (*P* = 0.007) were higher in MH lambs than those in M and MC lambs. The ruminal pH (*P* < 0.001) was lower in MC lambs than that in M and MH lambs. The concentration of ruminal NH_3_-N (*P* < 0.001) was higher in M lambs than that in MH and MC lambs. There was no significant difference (*P* > 0.05) in the concentrations of glucose, insulin, IGF-1, and BHBA in plasma among the three groups.

**Table 2:** The effect of alfalfa hay or concentrate starter on ruminal fermentation and blood parameter of pre-weaned lambs^1^

Item	M	MH	MC	SEM	*P*-value
Ruminal fermentation parameter					
pH	6.60^a^	6.85^a^	5.14^b^	0.17	< 0.001
Total VFA, mmol/L	8.36^c^	99.53^b^	174.38^a^	14.57	< 0.001
Acetate, mmol/L	4.21^c^	67.43^b^	94.60^a^	8.93	< 0.001
Propionate, mmol/L	2.20^c^	22.89^b^	43.50^a^	3.76	< 0.001
Isobutyrate, mmol/L	0.43^b^	0.92^a^	0.48^b^	0.06	< 0.001
Butyrate, mmol/L	0.81^c^	6.46^b^	29.34^a^	3.19	< 0.001
Isovalerate, mmol/L	0.55^b^	0.80^a^	0.51^b^	0.04	0.007
Valerate, mmol/L	0.17^c^	1.03^b^	5.95^a^	0.58	< 0.001
Acetate: propionate	2.00	3.05	2.24	0.20	0.069
NH_3_-N, mmol/L	11.45^a^	3.09^b^	4.41^b^	0.89	< 0.001
MCP, mg/mL	0.20^c^	0.81^b^	3.02^a^	0.29	< 0.001
Blood parameter					
Glucose, mmol/L	6.56	5.95	6.71	0.25	0.438
Insulin, μIU/mL	44.41	42.07	37.09	2.90	0.554
IGF-1, ng/mL	392.06	358.67	346.80	17.01	0.588
BHBA, mmol/L	0.31	0.47	0.58	0.05	0.125

^1^Values are shown as means ± pooled SEM, *n* = 8. Mean values within a column with unlike superscript letters were significantly different (*P* < 0.05). *M* goat milk group, *MH* goat milk plus alfalfa hay group, *MC* goat milk plus concentrate diet group, *VFA* volatile fatty acids, *MCP* microbial crude protein, *IGF-1* insulin-like growth factor 1, *BHBA* β-hydroxybutyric acid

### Rumen development parameters

As shown in Table 3, compared with lambs in M group, the lambs in MH and MC groups had greater emptied rumen weight (*P* < 0.001), emptied rumen weight/live body weight (*P* < 0.001), and thickness of rumen wall (*P* = 0.001), while there was no significant difference between MH and MC lambs. The rumen weight (*P* < 0.001) and thickness of muscles (*P* < 0.001) were highest in MH lambs, followed by MC lambs, and then M lambs. The rumen volume (*P* = 0.005) was greater in MH lambs than that in M lambs, and there was no significant difference of rumen volume between MH and MC lambs. The ruminal papilla length (*P* < 0.001), width (*P* < 0.001), ruminal epithelial absorption area (*P* < 0.001), and thickness of total epithelia (*P* < 0.001), stratum corneum (SC, *P* < 0.001), stratum granulosum (SG, *P* < 0.001), and stratum spinosum and basale (SS + SB, *P* < 0.001) were the highest in MC lambs, followed by MH lambs, and then M lambs. The density (*P* < 0.001) of ruminal papilla was greater in M lambs than that in MH and MC lambs.

**Table 3:** The effect of alfalfa hay or concentrate starter on rumen weight, volume, and ruminal papillae morphology of pre-weaned lambs^1^

Item	M	MH	MC	SEM	*P*-value
Rumen weight and volume					
Rumen weight, g	371.13^c^	1242.50^a^	749.63^b^	83.74	< 0.001
Emptied rumen weight, g	51.80^b^	154.00^a^	172.50^a^	11.72	< 0.001
Emptied rumen weight/live body weight, %	0.53^b^	1.80^a^	1.75^a^	0.13	< 0.001
Rumen volume, mL	716.25^b^	1528.75^a^	1199.38^ab^	111.12	0.005
Papillae morphology					
Length, mm	0.65^c^	1.79^b^	2.44^a^	0.07	< 0.001
Width, mm	0.49^c^	0.80^b^	1.32^a^	0.04	< 0.001
Density, n	310.00^a^	211.00^b^	158.00^b^	15.38	< 0.001
Surface, mm^2^/cm^2^	195.14^c^	577.69^b^	1006.69^a^	84.82	< 0.001
Thickness of different stratum					
Total epithelia, μm	90.78^c^	123.91^b^	216.03^a^	3.00	< 0.001
Stratum corneum, μm	13.42^c^	31.47^b^	48.78^a^	0.75	< 0.001
Stratum granulosum, μm	13.11^c^	17.64^b^	36.00^a^	0.42	< 0.001
Stratum spinosum and basale, μm	64.25^b^	74.81^b^	131.25^a^	2.43	< 0.001
Muscles layer, μm	742.73^c^	1006.08^a^	856.71^b^	20.46	< 0.001
Rumen wall, μm	1093.72^b^	1276.35^a^	1246.72^a^	22.86	0.001

^1^Values are shown as means ± pooled SEM, *n* = 8. Mean values within a column with unlike superscript letters were significantly different (*P* < 0.05). *M* goat milk group, *MH* goat milk plus alfalfa hay group, *MC* goat milk plus concentrate diet group

### Transcriptional profile in the rumen tissue

**Fig. 1:**
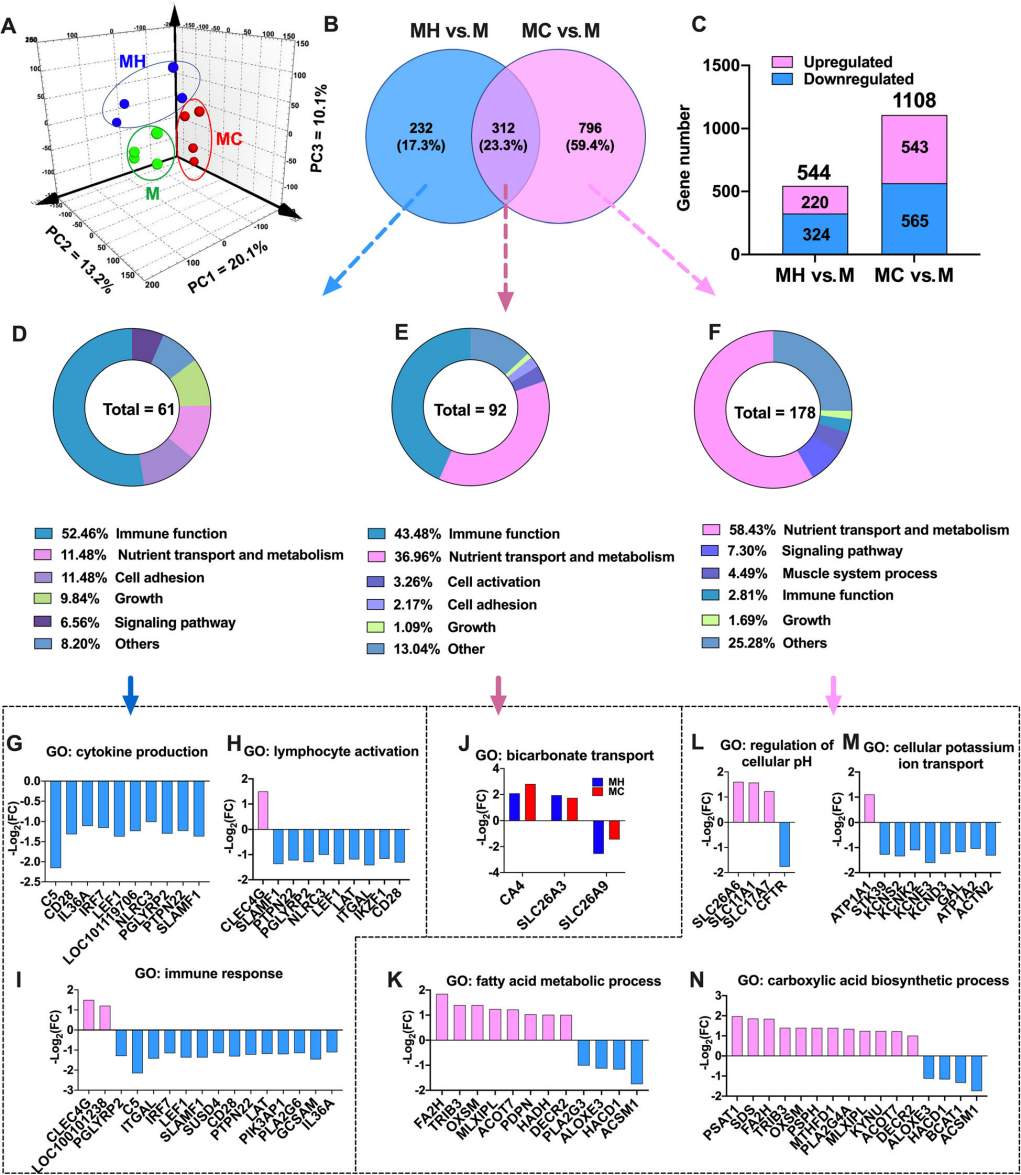
The PCA analysis of gene expressions, number of DEGs, biological process enrichment, and main DEGs in the rumen tissue. **a** The PCA analysis of gene expressions in the rumen tissue among three groups. **b** The number of unique and shared expression genes identified in the rumen tissue between “MH vs. M” and “MC vs. M”. **c** The number of DEGs in “MH vs. M” and “MC vs. M”. **d**-**f** The significant biological process classification of unique and shared DEGs. **g**-**i** Gene expressions in main biological terms of unique differentially expressed genes in MH vs. M. **j** Gene expressions in main biological terms of shared differentially expressed genes both in “MH vs. M” and “MC vs. M”. **k**-**n** Genes expression in mainly biological terms of unique DEGs in the MC vs. M. An adjusted *P* < 0.05 and an absolute value of [log_2_ (fold change)] > 1 were set as the filter criteria for significant differential expression genes (*n* = 4 per group)

A total of 501.81 million (41.82 ± 1.36 million reads per sample) high quality, paired reads generated from 12 rumen tissue samples, and the overall read alignment rate to the *Ovis aries* reference genome was 81% ± 2.22%. The principal components analysis (PCA) of total gene expression in rumen tissue samples demonstrated the marked clustering between 3 groups (Fig. 1a). There were 544 genes were significantly differentially expressed in the MH group compared with the M group, including 220 upregulated and 324 downregulated genes. One thousand one hundred and eight genes were significantly differentially expressed in the MC group compared with the M group, including 543 upregulated and 565 downregulated genes (Fig. 1c). As shown in Fig. 1b, Venn diagram showed there were 312 shared DEGs between the “MH vs. M” and “MC vs. M”, and 232 and 796 unique DEGs observed in the “MH vs. M” and “MC vs. M”, respectively.

### Functional analysis of 312 shared DEGs in the rumen tissue between “MH vs. M” and “MC vs. M”

A total of 92 biological process (BP) terms were significantly enriched out of which 43.48% were related to immune function, 36.96% were associated with nutrient transport and metabolism, 3.26% were linked to cell activation, while 2.17% were related to cell adhesion, and 1.09% to growth (Fig. 1e). The BP terms associated with immune function were divided into the classes of cytokine production, immune response, and immunocyte activation. The BP terms related to nutrient transport and metabolism were mainly divided into the classes of ion transport, lipid transport and metabolism, and nucleoside metabolic process (Supplemental Table 1). The DEGs were enriched in bicarbonate transport and ion transporter involved in VFA absorption including *CA4*, *SLC26A3*, and *SLC26A9* (Fig. 1j).

**Fig. 2:**
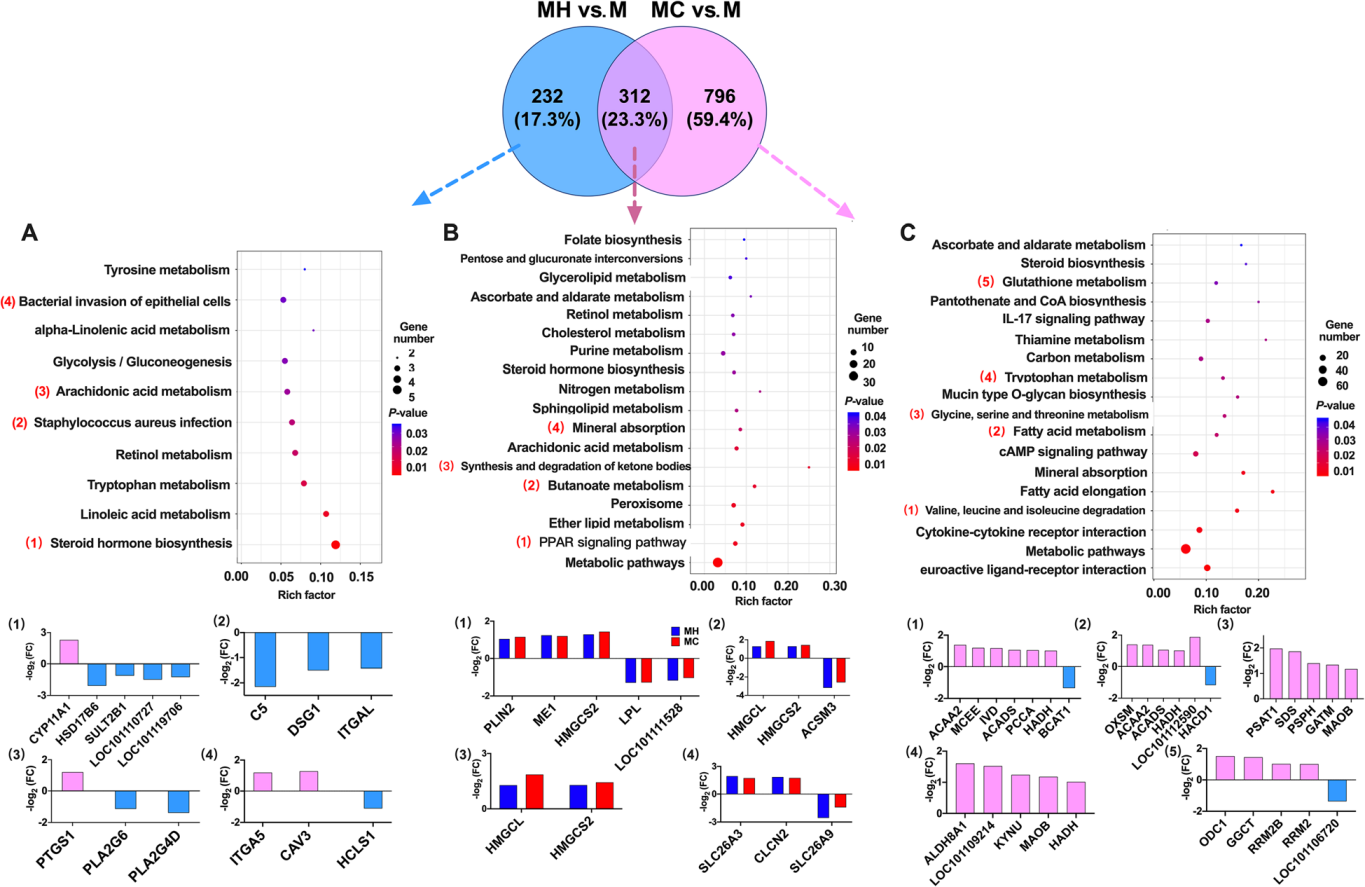
The KEGG pathways significantly enriched in the unique (**a** and **b**) and shared (**c**) DEGs identified in the rumen wall between “MH vs. M” and “MC vs. M”, and DEGs in main KEGG pathways. The significance of identified KEGG pathways was determined by *P* < 0.05

The results of KEGG pathway analysis showed that 18 pathways were significantly enriched (Fig. 2b). The pathways that were most enriched included metabolic pathways, PPAR signaling pathway (*PLIN2*, *ME1*, *HMGCS2*, *LPL*, and *LOC101111528*), butanoate metabolism (*HMGCL*, *HMGCS2*, and *ACSM3*), synthesis and degradation of ketone bodies (*HMGCL* and *HMGCS2*), and mineral absorption (*SLC26A3*, *CLCN2*, and *SLC26A9*) which is associated with VFA absorption and metabolism.

### Weighted gene co-expression network analysis of the correlation of host transcriptome with the rumen fermentation parameters and development indexes

**Fig. 3:**
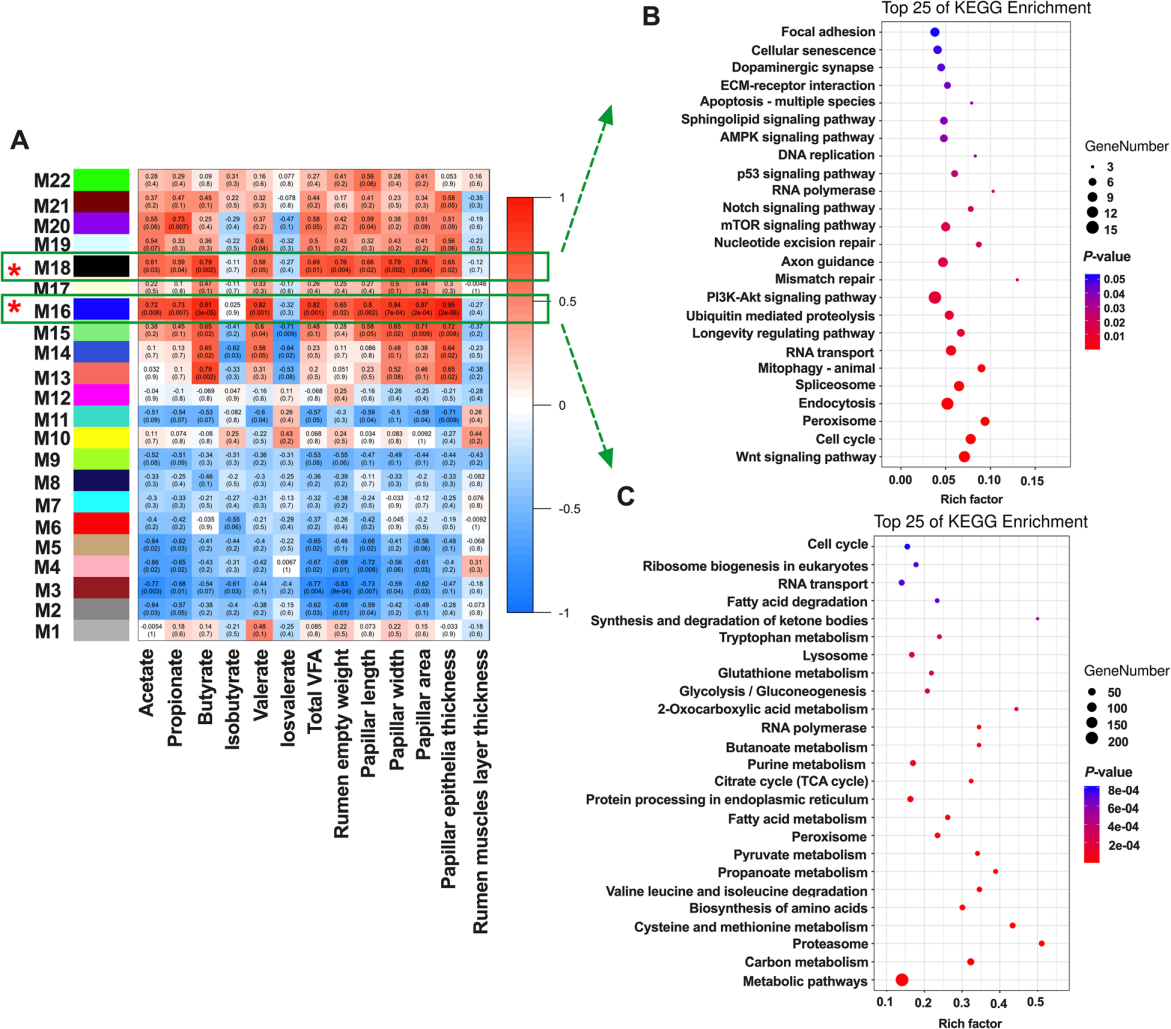
WGCNA identification of rumen tissue gene modules correlated with the rumen fermentation parameters and development indexes, and KEGG analysis of significant module. **a** WGCNA of the correlation of host transcriptome with the rumen fermentation parameters and development indexes. **b** Top KEGG pathway of genes significantly in the M16 module. **c** Top KEGG pathway of genes significantly in the M18 module. The significance of identified KEGG pathways was determined by *P* < 0.05. ECM, extracellular matrix; KEGG, Kyoto Encyclopedia of Genes and Genomes; VFA, volatile fatty acid; WGCNA, weighted gene co-expression network analysis. * *P* < 0.05, **P < 0.01

The use of WGCNA clustered the genes expressed in all rumen tissues into 22 gene modules (M1–M22). The two most significant modules, M16 (2,052 genes) and M18 (579 genes), showed a positive correlation with the rumen fermentation parameters and development indexes except for the concentration of isobutyrate and isovalerate (Fig. 3a). The BP terms of M16 and M18 were shown in Supplemental Table 2.

M16 module was related to many metabolism pathways, including carbon metabolism, cysteine and methionine metabolism, propanoate metabolism, pyruvate metabolism, fatty acid metabolism, citrate cycle, butanoate metabolism, glycolysis/gluconeogenesis, and synthesis and degradation of ketone bodies (Fig. 3c). The DEGs that were involved in these metabolism pathways including *FBP1*, *AHCYL2*, *TST*, *HMGCL*, *HMGCS2*, and *PCK2*, were significantly upregulated both in the MH and MC groups (Supplemental Table 3). M18 module was mainly enriched with the Wnt signaling pathway, cell cycle, PI3K-Akt signaling pathway, mTOR signaling pathway, Notch signaling pathway, p53 signaling pathway, DNA replication, and apoptosis—multiple species (Fig. 3b). Also, many genes involved in ruminal epithelial proliferation and apoptosis were discovered, including *TP53*, *CCND1*, *CCNB2*, *CASP3*, and *CASP8*.

### Function analysis of 223 DEGs observed in the rumen tissue of MC vs. MH

Two hundred and twenty-three genes were significantly differentially expressed in the MC group compared with the MH group, including 114 upregulated and 109 downregulated genes (Supplemental Fig. 2A). A total of 29 BP terms were significantly enriched out of which 17.24% were related to immune function, 17.24% were associated with nutrient transport and metabolism, 13.79% were linked to growth, 13.79% were associated response to stimulus, while 6.89% were related to muscle system process, and 3.45% to cell adhesion (Supplemental Fig. 2B). Six up-regulated genes (*CXCL8*, *IL36A*, *FABP4*, *IL1A*, *IL1B*, and *KRT1*) and 4 down-regulated genes (*NOS2*, *TSPAN2*, *SCN9A*, and *VNN1*) were enriched in the BP associated with inflammatory response. Four genes (*IL1A*, *IL36A, IL36RN*, and *EREG*) involved in the cytokine production were upregulated. Additionally, five DEGs (*GNMT*, *PSAT1*, *ARG1*, *ODC1*, and *SDS*) related to cellular amino acid metabolic process, were significantly downregulated, and seven genes (*GNMT*, *SCD*, *PSAT1*, *FABP4*, *ARG1*, *ODC1*, and *SDS*) associated with carboxylic acid metabolic process, were significantly upregulated (Supplemental Fig. 2D).

The results of KEGG pathway analysis showed that 27 pathways, mainly related to amino acid metabolism (biosynthesis of amino acids, glycine, serine and threonine metabolism, arginine and proline metabolism, and arginine biosynthesis.) and immune function (cytokine-cytokine receptor interaction, IL-17 signaling pathway, toll-like receptor signaling pathway, TNF signaling pathway, and viral protein interaction with cytokine and cytokine receptor), were significantly enriched (Supplemental Fig. 2C).

### Function analysis of 232 unique DEGs observed in the rumen tissue of MH vs. M

A total of 61 BP terms were significantly enriched out of which 52.46% were related to immune function while 11.48% were associated with nutrient transport and metabolism. Other BP terms affected include those related to cell adhesion (11.48%), growth (9.84%), and signaling pathway (6.56%) (Fig. 1d). The BP terms associated with immune function were divided into the classes of cytokine production, immune response, immunocyte activation, and the Toll-like receptor signaling pathway (Supplemental Table 4). The BP terms associated with cytokine production included regulation of cytokine production, cytokine production, and positive and negative regulation of cytokine production. Ten genes involved in the cytokine production were downregulated, including *C5*, *CD28*, *IL36A*, *IRF7*, *LEF1*, *LOC101119706*, *NLRC3*, *PGLYRP2*, *PTPN22*, and *SLAMF1* (Fig. 1g). Additionally, BP associated with immune response included immune effector process, immune response, regulation of immune system process, and regulation of immune response. Fourteen down-regulated DEGs (*C5*, *ITGAL*, *IRF7*, *LEF1*, *SLAMF1*, *SUSD4*, *CD28*, *PTPN22*, *LAT*, *PIK3AP1*, *PLA2G6*, *GCSAM*, and *IL36A*) and 2 up-regulated DGEs (*CLEC4G* and *LOC100101238*) were enriched in the BP associated with immune response (Fig. 1i). The BPs associated with immunocyte activation included leukocyte cell-cell adhesion, lymphocyte activation, leukocyte activation, and leukocyte differentiation. Nine down-regulated DEGs (*SLAMF1*, *PTPN22*, *PGLYRP2*, *NLRC3*, *LEF1*, *LAT*, *ITGAL*, *IKZF1*, and CD28) were enriched in the BP associated with lymphocyte activation (Fig. 1h).

The results of KEGG pathway analysis showed that 10 pathways were significantly enriched (Fig. 2a), including steroid hormone biosynthesis (*CYP11A1*, *HSD17B6*, *SULT2B1*, *LOC101110727*, and *LOC101119706*), *Staphylococcus aureus* infection (*C5*, *DSG1*, and *ITGAL*), arachidonic acid metabolism (*PTGS1*, *PLA2G6*, and *PLA2G4D*), and bacterial invasion of epithelial cells (*ITGA5*, *CAV3*, and *HCLS1*).

### Function analysis of 796 unique DEGs observed in the rumen tissue of MC vs. M

A total of 178 BP terms were significantly enriched out of which 58.43% were related to nutrient transport and metabolism while 7.30% were associated to signaling pathway. Other BP terms affected include those related to muscle system process (4.49%), immune function (2.81%), and growth (1.69%) (Fig. 1f). The BP terms related to nutrient transport and metabolism were mainly divided into the classes of ion transport, nitrogen transport and metabolism, and lipid transport and metabolism (Supplemental Table 5). The BPs associated with ion transport included cellular potassium ion transport, potassium ion transmembrane transport, negative regulation of ion transmembrane transporter activity, and regulation of intracellular pH. Four DEGs, *KCND3*, *KCNE3*, *KCNK2*, and *KCNS2*, in potassium transporter BP were significantly downregulated (Fig. 1m), and three genes, *SLC26A6*, *SLC11A1*, and *SLC17A7*, in regulation of intracellular pH, were significantly upregulated (Fig. 1l). The BPs associated with nitrogen transport and metabolism included nitrogen compound transport, carboxylic acid biosynthetic process, amide transport, and peptide transport. The genes expression belonging to the carboxylic acid biosynthetic process was shown in Fig. 1n. The BP associated with lipid transport and metabolism included fatty acid biosynthetic process, lipid metabolic process, positive regulation of lipid transport, fatty acid metabolic process, lipid transport, and so on. Eight up-regulated genes and 4 down-regulated genes enriched in the fatty acid metabolic process were shown in Fig. 1k.

The results of KEGG pathway analysis showed that 18 pathways were significantly enriched (Fig. 2c). Most KEGG pathways were associated with amino acid and fatty acid metabolisms, such as valine, leucine and isoleucine degradation (*ACAA2*, *MCEE*, *IVD*, *ACADS*, *PCCA*, *HADH*, and *BCAT1*), glycine, serine and threonine metabolism (*PSAT1*, *SDS*, *PSPH*, *GATM*, and *MAOB*), tryptophan metabolism (*ALDH8A1*, *LOC101109214*, *KYNU*, *MAOB*, and *HADH*), glutathione metabolism (*ODC1*, *GGCT*, *RRM2B*, *RRM2*, and *LOC101106720*), and fatty acid metabolism (*OXSM*, *ACAA2*, *ACADS*, *HADH*, *LOC101112590*, and *HACD1*).

## Discussion

In the present study, a 42-day-old liquid feeding lamb model was established to eliminate the effect of age on the experiment. Meanwhile, lambs in MH and MC groups were fed a uniform amount of liquid feed to ensure that a starter diet was the unique impact factor. Volatile fatty acids as energy production during its metabolism may directly stimulate rumen morphological development. Moreover, alfalfa hay introduction tended to affect the immune function of the rumen wall, while concentrate starter enhanced nutrient transport and metabolism function. These findings gained a comprehensive understanding of the regulatory mechanisms of the rumen wall morphological and functional development induced by different solid diet regimens, and provided new insights into the improvement of rumen wall development by nutritional strategies in young ruminants.

### Rumen fermentation and development

In the present study, compared with pure milk feeding, hay and concentrate starter did not affect ADG and BW of lambs, but the ADG of the MH lambs were lower than that in MC group. This could be explained by the lower ME contained in alfalfa hay than concentrate starter [26]. Compared with pure milk, alfalfa hay and concentrate introduction provides more carbon and nitrogen source for MCP and VFA synthesis in rumen [27, 28], which may explain the increase of rumen MCP and VFA concentrations with hay and concentrate starter introduction. Previous studies demonstrated that VFA was an important factor promoting rumen development [29]. The results of rumen morphological changes showed that alfalfa hay and concentrate starter introduction both positively affected rumen weight, volume, papillae surface, and thickness of rumen epithelium, which were consistent with previous studies [5, 10, 11, 30]. Previous studies indicated that hay introduction especially increased the volume of the rumen and thickness of rumen muscle in young ruminants, while concentrate starter introduction especially increased rumen weight and rumen papilla surface [9–11]. The largest volume and muscle layer thickness of rumen wall were found in the MH group, while the largest absorption and thickness of ruminal epithelia were found in the MC group.

### Molecular mechanisms involved in rumen wall morphological and functional development by transcriptome analysis

Three hundred and twelve shared DEGs, observed both in the MH vs. M and MC vs. M, may play a central role in promoting rumen wall morphological and functional development. These genes are mainly enriched in the biological processes of immune function and nutrient transport and metabolism, indicating that immune function and nutrient transport and metabolism play an important role in the development of the rumen. Meanwhile, immune function and nutrient transport and metabolism are the main functions of hay and concentrate, respectively, so these two parts are mainly discussed in the following specific functions of hay and concentrate. In the present study, the genes expression of *CA4* and *SLC26A3*, enriched in bicarbonate transport and ion transport, were increased both in the MH and MC group, and have been shown to participate in the ruminal absorption of VFA [31]. Moreover, the PPAR signaling pathway, butanoate metabolism, and synthesis and degradation of ketone bodies were found to be the most significantly enriched signal pathways in ruminal tissue with alfalfa hay or concentrate starter introduction, which are involved in VFA metabolism [32, 33]. Therefore, volatile fatty acid is a common medium for the development of rumen by hay and concentrate starter.

Weighted gene co-expression network analysis was conducted to further explore the relationship between VFA and rumen wall morphology. Two most significant modules, M16 and M18, were found in the present study. During BP analysis of genes in the M16 module, up-regulated organic acid metabolic processes and ATP metabolic biological processes were highlighted as the characteristics of rumen wall development. Meanwhile, increased carbon metabolism, propanoate metabolism, pyruvate metabolism, citrate cycle, butanoate metabolism, and synthesis and degradation of ketone bodies pathways were also found as features of rumen wall development. These results were partly consistent with Naeem et al. who not only indicated that the metabolism of pyruvate, VFA, and long-chain fatty acids, were increased in rumen epithelia of the calf in response to enhanced plane of nutrition but also clarified that PPAR may be an important signal pathway to promote rumen metabolism and development [32]. Similarly, during the analysis of shared DEGs, the PPAR signaling pathway was the most significantly changed pathway induced by alfalfa hay or concentrate starter. Previous studies indicated that the mitotic process is a highly endoenergetic process, and important in promoting rumen epithelial development and growth, especially G_1_ period [34–36]. Interestingly, the expression of *CCNE*, enriched in the cell cycle pathway, was increased in the rumen tissue of the MH and MC group. Specifically, *CCNE* stability may depend on mitochondrial ATP producing capacity [37]. Therefore, stabilization of *CCNE* during G_1_-S transition may be caused by increased VFA metabolism after solid diet introduction. In addition, the genes in M18 are mainly enriched in signal transduction, including Wnt signaling pathway, PI3K-Akt signaling pathway, mTOR signaling pathway, Notch signaling pathway, p53 signaling pathway, and AMPK signaling pathway. These signaling pathways were closely related to cell proliferation, apoptosis, and differentiation [36, 38–41]. However, the main genes enriched in the KEGG pathway of signal transduction were not affected by solid diet introduction. Therefore, the energy production during VFA metabolism may drive the rumen wall development directly, and the PPAR signaling pathway may be the link between VFA metabolism and rumen development.

### The different molecular and biological processes involved in rumen wall morphological and functional development between hay and concentrate starter introduction

In the present study, two hundred and twenty-three genes were differentially expressed in the MC vs. MH group. The mainly enriched biological process were related to the immune function and nutrient transport and metabolism of rumen tissue. Here, the expression of genes related to interleukins production were lower in MH than MC, including *IL1A*, *IL1B*, *IL36A*, *IL36RN*, *IL36G*, and *IL12RB2*. These genes also enriched in the pathway of cytokine-cytokine receptor interaction, IL-17 signaling pathway, Toll-like receptor signaling pathway, TNF signaling pathway, and viral protein interaction with cytokine receptor. The decreased expression of immune-related genes (including *ILs*, *TNF*, and *TLRs*) has been reported in rumen tissue of lambs when fed solid diet [42, 43]. These results suggested there is greater resistance to inflammation within the rumen tissue in MH than MC lambs. Furthermore, compared with MH lambs, the upregulated genes (*GNMT*, *PSAT1*, *ARG1*, *ODC1* and *SDS*) in MC lambs, were involved in cellular amino acid metabolic process, carboxylic acid metabolic process, and the pathway of many amino acid metabolism, indicating enhanced amino acid metabolism in the rumen tissue. These results indicated that hay and concentrate starter exert distinct influences on the rumen wall functional development in pre-weaned lambs. Thus, in the present study, the specific biological processes upon hay or concentrate starter regimes were further investigated.

### The specific molecular and biological processes involved in rumen wall morphological and functional development by hay introduction

To clarify the special functional genes and biological processes when alfalfa hay was introduced, the analysis of the biological functions of the 232 unique DEGs (MH vs. M) were performed. Interestingly, the most enriched biological process was related to the immune function of rumen tissue. According to our findings, alfalfa hay introduction downregulated the cytokine production process. The down-regulated DEGs of *IL36A*, *CD28*, *SLAMF1*, *LEF1*, and *PGLYRP2* reduced the production of pro-inflammatory factors, such as IL36, IL-2, TNF-α, IL-1β, and IL-6 [44–48]. Moreover, the unique up-regulated gene (*CLEC4G*) and down-regulated genes (*ITGAL* and *LAT*) in the MH group were enriched in immune response and lymphocyte activation, which may reduce immune and inflammatory responses in rumen tissue [49–51]. Previous studies showed that high VFA concentration decreased the expression of genes involved in cholesterol biosynthesis in the rumen epithelia [11, 52]. Similarly, in the present study, the cholesterol decomposition process in steroid hormone biosynthesis was downregulated by alfalfa hay introduction, which may be explained by high VFA production in the rumen. In addition, alfalfa hay introduction also downregulated the arachidonic acid production process, proved by decreased *PLA2G4* and *PLA2G6*. It is well established that cholesterol and arachidonic acid regulate cellular inflammation, oxidative stress, proliferation, and membrane permeability [52, 53], which may affect the rumen immune function. Previous studies indicated that ruminal epithelial cells recognize microbial components through Toll-like receptors and PGLYRPs [1, 54]. The decreased *PGLYRP2* expression in MH may be resulted from the establishment of microbial immune tolerance and important for minimizing harmful inflammatory responses [1, 54]. In the present study, the unique DEGs in MH significantly enriched in the bacterial invasion of epithelial cells and *Staphylococcus aureus* infection pathway. In these DEGs, *ITGA5* and *CAV3* can effectively prevent pathogens from invading host cells [55] and regulate host immune response to the majority of bacterial invasion by promoting bacterial clearance [56], respectively. *DSG1* is the specific receptor for exfoliative toxin A cleavage and essential for the promotion of staphylococcus adhesion to host cells [57]. Moreover, the decreased expression of the lipopolysaccharide binding protein (*LBP*) was also observed in alfalfa hay supplementation. Taking these results together, hay supplementation may be beneficial to maintain the healthy rumen development. Thus, future studies are needed to focus on the bacteria structure as well as the concentration of lipopolysaccharide in the rumen content and epithelium to define their roles in affecting rumen tissue gene expression and its relationship with immune response.

### The specific molecular and biological processes involved in rumen wall morphological and functional development by concentrate starter introduction

The biological functions analysis of the 796 unique DEGs (MC vs. M) showed that concentrate starter introduction especially affected the nutrient transport and metabolism in rumen tissue. The unique up-regulated *SLC26A6* in the MC group involved in the regulation of cellular pH has been extensively studied in rumen VFA absorption [58]. Moreover, lower expression of *KCND3*, *KCNE3*, *KCNK2*, and *KCNS2* enriched in cellular potassium ion transport and potassium ion transmembrane transport were suggestive of lower ammonia absorption in the rumen epithelia of lambs fed concentrate starter [59, 60]. This may be the reason of low NH_3_-N concentration in the rumen of lambs fed concentrate starter. Previous study indicated that ammonia reduced the rate of endogenous urea transfer to the lumen of the gastrointestinal tract [61]. Likewise, enrichment in the nitrogen compound transport process was observed as well as a significant increase in the expression of *SLC14A1*, formerly known as urea transfer protein [62], which suggested increased transport of urea from the blood into the rumen. Therefore, these results demonstrated that concentrate starter introduction increased the rumen urea-N recycling and nitrogen utilization. It is of note that we also found many signaling pathways involved in the amino acid metabolism. Previous studies indicated that a comparatively low amount of amino acids and peptides may be absorbed and metabolized by the rumen tissue [63–65]. Moreover, the development of a rumen wall needs increased cell and protein turnover [66–68]. Previous studies showed that amino acids were absorbed by the animal host in the intestine and transferred to organ tissues for protein synthesis and oxidation [69, 70]. Taking these results together, we speculated that the synthesis of MCP in the rumen promoted protein turnover and oxidation in the rumen tissue, no matter where it was absorbed (rumen or the intestine). In the present study, 6 upregulated DEGs were annotated in the valine, leucine, and isoleucine degradation. Meanwhile, the glycine, serine and threonine metabolism and tryptophan metabolism were upregulated in concentrate starter introduction. These metabolites are the substrates of the citric acid cycle, generating energy-containing compounds, including NADH, FADH, and ATP. They are responsible for the activation of some biological processes and might further promote rumen wall development [14]. Fatty acids are essential metabolic fuels and are vital for cell proliferation in cellular membranes [32]. In the present study, many unique DEGs were annotated in fatty acid metabolism and fatty acid elongation upon the introduction of concentrate starter, which was beneficial to the proliferation and turnover of rumen epithelial cells. Therefore, the above results provide evidence for the critical role of the nutrient absorption and metabolism in the rumen wall functional development with concentrate starter introduction.

## Conclusions

**Fig. 4:**
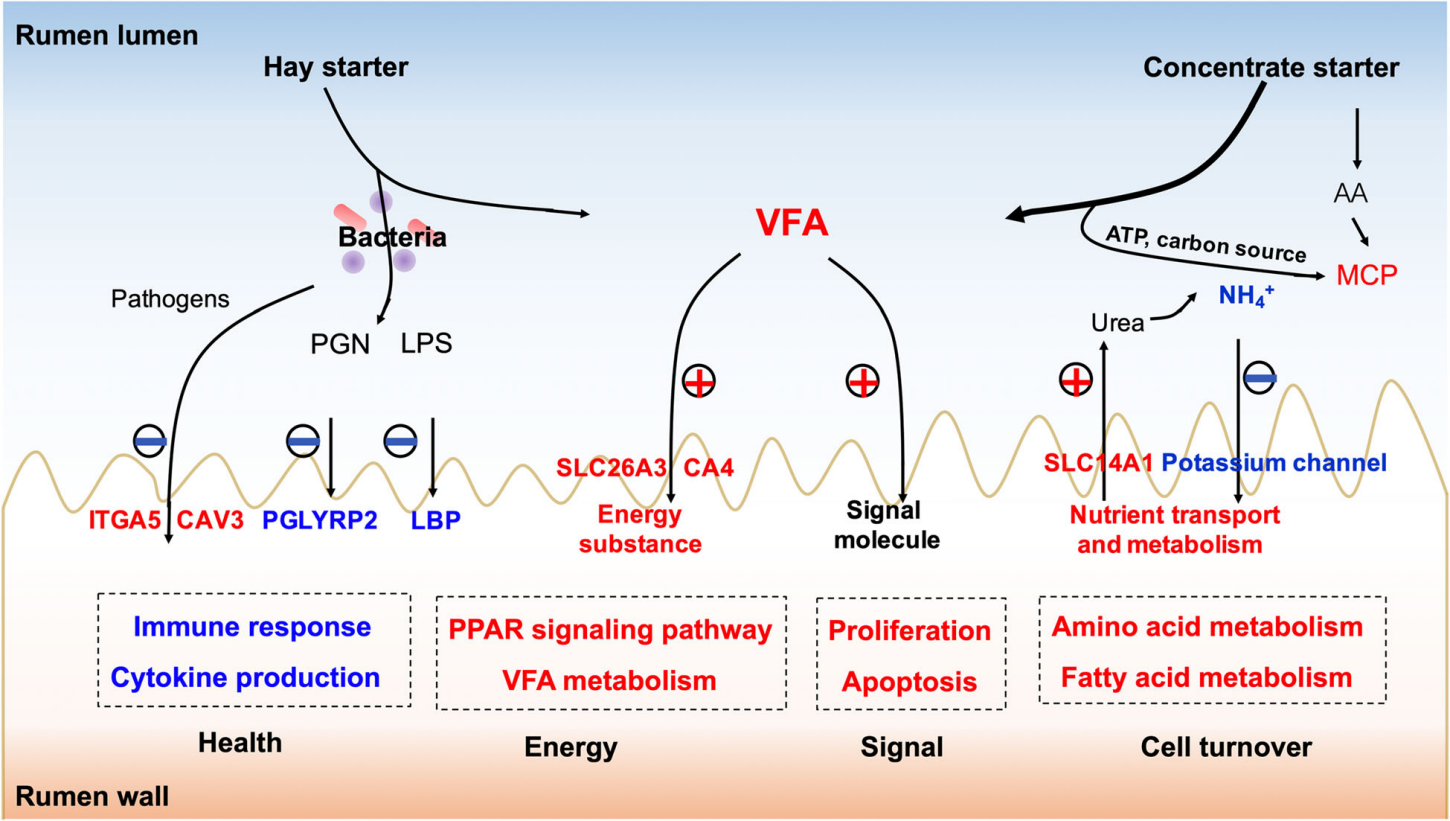
Summary of clarifying how different solid diet regimens (hay or concentrate) drive rumen wall development and characterize their shared and unique biological process between “MH vs. M” and “MC vs. M”. The plus sign represents upregulated or activation, and the minus sign represents downregulated or inhibition. AA, amino acid; LPS, lipopolysaccharide; LBP, LPS binding protein; MCP, microbial crude protein; VFA, volatile fatty acid

In summary, VFA plays a major role in promoting rumen development, as energy production during VFA metabolism may directly stimulate rumen development. Moreover, the hay/concentrate starter drives rumen wall morphological and functional development in the special biological process of pre-weaning lambs. Alfalfa hay introduction primarily enhanced the ability of rumen epithelium to resist bacterial invasion and facilitated establishment of the immune function in the rumen wall of pre-weaned lambs. While, concentrate starter introduction was likely to promote the nutrient metabolism function and increased amino acid and fatty acid metabolism in the rumen tissue (Fig. 4). The functions of immune metabolism in the rumen wall are closely related to animal health and production performance. Therefore, in ruminant production, to promote efficient and healthy animal performance, the special impacts of hay and concentrate starter introduction on the morphological and functional development of the rumen wall should be fully considered.

## Supplementary Information

**Additional file 1: Supplemental Table 1**. The main biological process enrichment analysis of shared DEGs both in MH and MC group lambs. **Supplemental Table 2**. Top 20 biological process terms significantly enriched in the M16 and M18 module. **Supplemental Table 3**. Top 25 KEGG pathway terms significantly enriched in the M16 and M18 module. **Supplemental Table 4**. The BP terms associated with immune function were enriched from unique DEGs observed in the MH vs. M. **Supplemental Table 5**. The BP terms associated with nutrient transport and metabolism were enriched from unique DEGs observed in the MC vs. M. **Supplemental Fig. 1**. Effect of alfalfa hay or concentrate starter on the ADFI (A), ADG (B), and BW (C) of pre-weaned lambs. Values are shown as means ± pooled SEM, *n* = 8. Mean values within a column with unlike superscript letters were significantly different (*P* < 0.05). M, goat milk group; MH, goat milk plus alfalfa hay group; MC, goat milk plus concentrate diet group; ADFI, average daily feed intake; ADG, average daily gain; BW, body weight. **Supplemental Fig. 2**. The comparison of MC and MH in transcriptome analysis. A. The number of DEGs identified in MC vs MH. B. The significant biological process classification of DEGs. C. The significantly enriched KEGG pathways of DEGs. D. Genes expression in mainly biological terms of DEGs. E. Genes expression in mainly KEGG pathways of DEGs. An adjusted *P* < 0.05 and an absolute value of [log_2_ (fold change)] > 1 were set as the filter criteria for significant differential expression genes (*n* = 4 per group). The significance of identified KEGG pathways was determined by *P* < 0.05

## Data Availability

The datasets used and analyzed during the current study are available from the corresponding author on reasonable request.

## Abbreviations

AHCYL2: Adenosylhomocysteinase like 2
BHBA: β-Hydroxybutyric acid
BP: Biological process
BW: Body weight
CA4: Carbonic anhydrase 4
CASPs: Caspases
CCNs: Cyclins
DEGs: Differentially expressed genes
FBP1: Fructose-bisphosphatase 1
FPKM: Fragments per kilobase of transcript per million fragments mapped
GPRs: G protein-coupled receptors
HDACs: Histone deacetylases
HMGCL: 3-Hydroxy-3-methylglutaryl-CoA lyase
HMGCS2: 3-Hydroxy-3-methylglutaryl CoA synthase 2
KCND3: Potassium voltage-gated channel subfamily D member 3
KCNE3: Potassium voltage-gated channel subfamily E member 3
KCNK2: Potassium voltage-gated channel subfamily K member 2
KCNS2: Potassium voltage-gated channel subfamily S member 2
KEGG: Kyoto Encyclopedia of Genes and Genomes
M: Goat milk powder only
MC: Goat milk powder + concentrate starter
MCP: Microbial crude protein
ME: Metabolic energy
MH: Goat milk powder + alfalfa hay
NH_3_-N: Ammonia nitrogen
PCA: Principal components analysis
PCK2: Phosphoenolpyruvate carboxykinase 2
PGLYRP2: Peptidoglycan recognition proteins 2
PLA2G4: Phospholipase A2 group 4
SLC11A1: Solute carrier family 11 member 1
SLC17A7: Solute carrier family 17 member 7
SLC26A6: Solute carrier family 26 member 6
TP53: Tumor protein p53
TST: Thiosulfate sulfurtransferase
VFAs: Volatile fatty acids
WGCNA: Weighted gene co-expression network analysis
