# Supplementary material for: Transcriptomic analysis reveals the molecular mechanisms of rumen wall morphological and functional development induced by different solid diet introduction in a lamb model
Source: J Anim Sci Biotechnol. 2021 Mar 10;12:33. doi: 10.1186/s40104-021-00556-4 (PMC7944623; doi:10.1186/s40104-021-00556-4)
Supplement: Supplementary file 1 — Additional file 1: Supplemental Table 1. The main biological process enrichment analysis of shared DEGs both in MH and MC group lambs. Supplemental Table 2. Top 20 biological process terms significantly enriched in the M16 and M18 module. Supplemental Table 3. Top 25 KEGG pathway terms significantly enriched in the M16 and M18 module. Supplemental Table 4. The BP terms associated with immune function were enriched from unique DEGs observed in the MH vs. M. Supplemental Table 5. The BP terms associated with nutrient transport and metabolism were enriched from unique DEGs observed in the MC vs. M. Supplemental Fig. 1. Effect of alfalfa hay or concentrate starter on the ADFI (A), ADG (B), and BW (C) of pre-weaned lambs. Values are shown as means ± pooled SEM, n = 8. Mean values within a column with unlike superscript letters were significantly different (P < 0.05). M, goat milk group; MH, goat milk plus alfalfa hay group; MC, goat milk plus concentrate diet group; ADFI, average daily feed intake; ADG, average daily gain; BW, body weight. Supplemental Fig. 2. The comparison of MC and MH in transcriptome analysis. A. The number of DEGs identified in MC vs MH. B. The significant biological process classification of DEGs. C. The significantly enriched KEGG pathways of DEGs. D. Genes expression in mainly biological terms of DEGs. E. Genes expression in mainly KEGG pathways of DEGs. An adjusted P < 0.05 and an absolute value of [log2 (fold change)] > 1 were set as the filter criteria for significant differential expression genes (n = 4 per group). The significance of identified KEGG pathways was determined by P < 0.05 [file 40104_2021_556_MOESM1_ESM.docx]

**Supplemental Table 1**. The mainly biological process enrichment analysis of shared DEGs both in MH and MP group lambs.

| Function | ID | Biological term | Number of genes | *P*-value |
| --- | --- | --- | --- | --- |
| Cytokine production | |  |  |  |
|  | GO:0001816 | Cytokine production | 15 | 0.002 |
|  | GO:0001817 | Regulation of cytokine production | 14 | 0.002 |
|  | GO:0032609 | Interferon-gamma production | 6 | 0.004 |
|  | GO:0001819 | Positive regulation of cytokine production | 10 | 0.008 |
|  | GO:0032649 | Regulation of interferon-gamma production | 5 | 0.015 |
|  | GO:0002370 | Natural killer cell cytokine production | 2 | 0.027 |
|  | GO:0002727 | Regulation of natural killer cell cytokine production | 2 | 0.027 |
|  | GO:0032729 | Positive regulation of interferon-gamma production | 4 | 0.029 |
| Immune response | |  |  |  |
|  | GO:0002429 | Immune response-activating cell surface receptor signaling pathway | 8 | 0.000 |
|  | GO:0002768 | Immune response-regulating cell surface receptor signaling pathway | 8 | 0.000 |
|  | GO:0002757 | Immune response-activating signal transduction | 10 | 0.001 |
|  | GO:0002764 | Immune response-regulating signaling pathway | 10 | 0.001 |
|  | GO:0002253 | Activation of immune response | 10 | 0.001 |
|  | GO:0006955 | Immune response | 20 | 0.002 |
|  | GO:0050776 | Regulation of immune response | 13 | 0.005 |
|  | GO:0050778 | Positive regulation of immune response | 11 | 0.007 |
|  | GO:0002697 | Regulation of immune effector process | 10 | 0.010 |
|  | GO:0002684 | Positive regulation of immune system process | 14 | 0.014 |
|  | GO:0002699 | Positive regulation of immune effector process | 6 | 0.016 |
|  | GO:0002682 | Regulation of immune system process | 19 | 0.017 |
|  | GO:0002700 | Regulation of production of molecular mediator of immune response | 5 | 0.022 |
|  | GO:0002440 | Production of molecular mediator of immune response | 6 | 0.022 |
|  | GO:0007159 | Leukocyte cell-cell adhesion | 10 | 0.024 |
|  | GO:0002702 | Positive regulation of production of molecular mediator of immune response | 4 | 0.032 |
|  | GO:0002252 | Immune effector process | 12 | 0.034 |
|  | GO:0002715 | Regulation of natural killer cell mediated immunity | 3 | 0.036 |
| Immunocyte activation | |  |  |  |
|  | GO:0045321 | Leukocyte activation | 15 | 0.004 |
|  | GO:0070486 | Leukocyte aggregation | 10 | 0.015 |
|  | GO:0048535 | Lymph node development | 3 | 0.019 |
|  | GO:0046649 | Lymphocyte activation | 12 | 0.020 |
|  | GO:0046651 | Lymphocyte proliferation | 7 | 0.026 |
|  | GO:0050851 | Antigen receptor-mediated signaling pathway | 5 | 0.026 |
|  | GO:0032943 | Mononuclear cell proliferation | 7 | 0.026 |
|  | GO:0030098 | Lymphocyte differentiation | 8 | 0.029 |
|  | GO:0002696 | Positive regulation of leukocyte activation | 7 | 0.031 |
|  | GO:0070661 | Leukocyte proliferation | 7 | 0.032 |
|  | GO:0042110 | T cell activation | 9 | 0.034 |
|  | GO:0070489 | T cell aggregation | 9 | 0.034 |
|  | GO:0071593 | Lymphocyte aggregation | 9 | 0.034 |
|  | GO:0002694 | Regulation of leukocyte activation | 9 | 0.035 |
| Ion transport | |  |  |  |
|  | GO:0015711 | Organic anion transport | 8 | 0.000 |
|  | GO:0015701 | Bicarbonate transport | 3 | 0.001 |
|  | GO:0006820 | Anion transport | 8 | 0.001 |
|  | GO:0006811 | Ion transport | 16 | 0.005 |
|  | GO:0098771 | Inorganic ion homeostasis | 10 | 0.026 |
|  | GO:0070838 | Divalent metal ion transport | 7 | 0.031 |
|  | GO:0072511 | Divalent inorganic cation transport | 7 | 0.031 |
|  | GO:0050801 | Ion homeostasis | 10 | 0.042 |
|  | GO:0055067 | Monovalent inorganic cation homeostasis | 4 | 0.042 |
| Lipid transport and metabolism | | |  |  |
|  | GO:0044255 | Cellular lipid metabolic process | 22 | 0.000 |
|  | GO:0006629 | Lipid metabolic process | 24 | 0.000 |
|  | GO:0010876 | Lipid localization | 10 | 0.001 |
|  | GO:0006641 | Triglyceride metabolic process | 5 | 0.003 |
|  | GO:0006869 | Lipid transport | 8 | 0.004 |
|  | GO:0008610 | Lipid biosynthetic process | 11 | 0.006 |
|  | GO:0006639 | Acylglycerol metabolic process | 5 | 0.006 |
|  | GO:0006638 | Neutral lipid metabolic process | 5 | 0.006 |
|  | GO:0006644 | Phospholipid metabolic process | 8 | 0.014 |
|  | GO:0046486 | Glycerolipid metabolic process | 7 | 0.021 |
|  | GO:0019915 | Lipid storage | 4 | 0.024 |
|  | GO:0015908 | Fatty acid transport | 4 | 0.026 |
| Nucleoside metabolic process | | |  |  |
|  | GO:0046128 | Purine ribonucleoside metabolic process | 7 | 0.023 |
|  | GO:0042278 | Purine nucleoside metabolic process | 7 | 0.024 |
|  | GO:0009119 | Ribonucleoside metabolic process | 7 | 0.028 |
|  | GO:1901068 | Guanosine-containing compound metabolic process | 3 | 0.033 |
|  | GO:0009116 | Nucleoside metabolic process | 7 | 0.038 |
|  | GO:0009132 | Nucleoside diphosphate metabolic process | 4 | 0.042 |
| Organic acid | |  |  |  |
|  | GO:0046942 | Carboxylic acid transport | 5 | 0.030 |
|  | GO:0015718 | Monocarboxylic acid transport | 4 | 0.037 |
| Nitrogen transport and metabolism | | |  |  |
|  | GO:0071705 | Nitrogen compound transport | 9 | 0.034 |
| Others |  |  |  |  |
|  | GO:0019637 | organophosphate metabolic process | 17 | 0.003 |
|  | GO:0042592 | homeostatic process | 22 | 0.016 |
|  | GO:0048878 | chemical homeostasis | 14 | 0.029 |
|  | GO:1901657 | glycosyl compound metabolic process | 7 | 0.044 |

**Supplemental Table 2. Top 20 biological process terms significantly enriched in the M16 and M18 module.**

| Module | ID | Biological term | Number of gene | *P*-value |
| --- | --- | --- | --- | --- |
| M16 |  |  |  |  |
|  | GO:0006082 | Organic acid metabolic process | 112 | 5.71E-04 |
|  | GO:0043436 | Oxoacid metabolic process | 103 | 1.29E-11 |
|  | GO:0019752 | Carboxylic acid metabolic process | 102 | 2.26E-11 |
|  | GO:0009161 | Ribonucleoside monophosphate metabolic process | 40 | 1.79E-07 |
|  | GO:0009167 | Purine ribonucleoside monophosphate metabolic process | 39 | 1.83E-07 |
|  | GO:0009123 | Nucleoside monophosphate metabolic process | 41 | 2.13E-07 |
|  | GO:0009126 | Purine nucleoside monophosphate metabolic process | 39 | 2.28E-07 |
|  | GO:0006753 | Nucleoside phosphate metabolic process | 75 | 3.76E-07 |
|  | GO:0009119 | Ribonucleoside metabolic process | 45 | 7.64E-07 |
|  | GO:0009117 | Nucleotide metabolic process | 72 | 1.01E-06 |
|  | GO:0032787 | Monocarboxylic acid metabolic process | 60 | 1.14E-06 |
|  | GO:0046128 | Purine ribonucleoside metabolic process | 43 | 1.28E-06 |
|  | GO:1901657 | Glycosyl compound metabolic process | 48 | 1.35E-06 |
|  | GO:0042278 | Purine nucleoside metabolic process | 43 | 1.82E-06 |
|  | GO:0055086 | Nucleobase-containing small molecule metabolic process | 77 | 2.05E-06 |
|  | GO:0009116 | Nucleoside metabolic process | 46 | 2.66E-06 |
|  | GO:0046034 | ATP metabolic process | 33 | 3.58E-06 |
|  | GO:0009141 | Nucleoside triphosphate metabolic process | 39 | 7.24E-06 |
|  | GO:0009144 | Purine nucleoside triphosphate metabolic process | 36 | 7.83E-06 |
|  | GO:1901566 | Organonitrogen compound biosynthetic process | 130 | 2.23E-05 |
| M18 |  |  |  |  |
|  | GO:0000278 | Mitotic cell cycle | 34 | 6.99E-06 |
|  | GO:1903047 | Mitotic cell cycle process | 29 | 3.97E-05 |
|  | GO:0007059 | Chromosome segregation | 20 | 4.45E-05 |
|  | GO:0007049 | Cell cycle | 49 | 8.07E-05 |
|  | GO:0051301 | Cell division | 18 | 1.59E-04 |
|  | GO:0022402 | Cell cycle process | 42 | 1.72E-04 |
|  | GO:2000369 | Regulation of clathrin-mediated endocytosis | 5 | 3.41E-04 |
|  | GO:0000724 | Double-strand break repair via homologous recombination | 9 | 5.15E-04 |
|  | GO:0007346 | Regulation of mitotic cell cycle | 21 | 5.71E-04 |
|  | GO:0000725 | Recombinational repair | 9 | 5.82E-04 |
|  | GO:0051649 | Establishment of localization in cell | 54 | 5.89E-04 |
|  | GO:0007067 | Mitotic nuclear division | 16 | 6.66E-04 |
|  | GO:0051726 | Regulation of cell cycle | 34 | 7.57E-04 |
|  | GO:0007034 | Vacuolar transport | 15 | 1.93E-03 |
|  | GO:0007033 | Vacuole organization | 12 | 2.15E-03 |
|  | GO:0046907 | Intracellular transport | 42 | 3.51E-03 |
|  | GO:0016197 | Endosomal transport | 13 | 3.67E-03 |
|  | GO:0000070 | Mitotic sister chromatid segregation | 10 | 4.20E-03 |
|  | GO:0099537 | Trans-synaptic signaling | 17 | 4.64E-03 |
|  | GO:0007268 | Chemical synaptic transmission | 17 | 4.64E-03 |

**Supplemental** **Table 3. Top 25 KEGG pathway terms significantly enriched in the M16 and M18 module.**

| Module | Term | *P*-value | DEGs in MH | DEGs in MC |
| --- | --- | --- | --- | --- |
| M16 |  |  |  |  |
|  | Metabolic pathways | 7.31E-24 | PCK2, IMPDH1, FBP1, CERS4, AHCYL2, HMGCL, TST, ISYNA1, NQO1, BLVRB, AMPD3, HMGCS2, ADK, CMBL, PDE6A, CA12, PAPSS2, CA4 | PCK2, TM7SF2, PCCA, IMPDH1, FBP1, CERS4, GLO1, ENTPD3, ENTPD2, GPX2, HMGCL, GALNT15, MIF, NFS1, ATP6V1A, GGCT, TST, ISYNA1, NT5E, GATM, ACOT8, NQO1, BLVRB, IVD, HADH, MTHFD1, MAOB, ESD, PDE6D, EPHX2, AMPD3, ACADS, OXSM, HMGCS2, UGDH, GALM, ADK, CYP1A1, CMBL, PSPH, GPT2, ADA, ST6GAL2, PCBD1, RRM2, ODC1, UGT1A3, KYNU, PDE6A, MCEE, CA12, PAPSS2, FPGS, PLA2G4A, CA9, SDS, CA4, PSAT1, RDH12 |
|  | Carbon metabolism | 1.49E-14 | FBP1 | GPT2 PSAT1, PSPH, SDS, ACADS, MCEE, PCCA, ESD, FBP1, ME2 |
|  | Proteasome | 6.23E-12 | - | PSMA5 |
|  | Cysteine and methionine metabolism | 8.23E-11 | AHCYL2, TST | TST, PSAT1, SDS |
|  | Biosynthesis of amino acids | 5.02E-08 | - | GPT2, PSAT1, PSPH, SDS |
|  | Valine leucine and isoleucine degradation | 1.49E-07 | HMGCL, HMGCS2 | HMGCL, HMGCS2, ACADS, HADH, IVD, MCEE, PCCA |
|  | Propanoate metabolism | 1.17E-06 | - | ACADS, MCEE, PCCA |
|  | Pyruvate metabolism | 4.06E-06 | PCK2 | PCK2, GLO1, ME2 |
|  | Peroxisome | 5.27E-06 | CRAT, HMGCL | ACOT8, CRAT, DECR2, EPHX2, HMGCL, PECR |
|  | Fatty acid metabolism | 8.12E-06 | - | ACADS, HADH, OXSM |
|  | Protein processing in endoplasmic reticulum | 3.84E-05 | - | - |
|  | Citrate cycle (TCA cycle) | 6.53E-05 | PCK2 | PCK2 |
|  | Purine metabolism | 7.25E-05 | ADK, AMPD3, IMPDH1, PAPSS2, PDE6A | RRM2, ADA, ADK, AMPD3, ENTPD2, ENTPD3, IMPDH1, NT5E, PAPSS2, PDE6A, PDE6D |
|  | Butanoate metabolism | 9.04E-05 | HMGCL, HMGCS2 | HMGCL, HMGCS2, ACADS, HADH |
|  | 2-Oxocarboxylic acid metabolism | 1.14E-04 | - | GPT2 |
|  | Glycolysis / Gluconeogenesis | 2.46E-04 | FBP1, PCK2 | FBP1, PCK2, GALM |
|  | Glutathione metabolism | 2.54E-04 | - | GGCT, GPX2, ODC1, RRM2 |
|  | Lysosome | 2.57E-04 | - | ARSG, ABCB9, CTSL, DNASE2, LIPA, SLC11A1 |
|  | Tryptophan metabolism | 3.35E-04 | - | CYP1A1, HADH, KYNU, MAOB |
|  | Synthesis and degradation of ketone bodies | 5.16E-04 | HMGCL, HMGCS2 | HMGCL, HMGCS2 |
|  | Fatty acid degradation | 7.03E-04 | - | ACADS, HADH |
|  | Cell cycle | 8.02E-04 | CCNE1 | CCNE1, CDKN1A, ORC1 |
| M18 |  |  |  |  |
|  | Peroxisome | 3.71E-04 | NUDT7 | NUDT7 |
|  | PI3K-Akt signaling pathway | 1.21E-02 | TNC | TNC |
|  | ECM-receptor interaction | 4.20E-02 | TNC | TNC |
|  | Dopaminergic synapse | 4.65E-02 | - | CALY |
|  | Focal adhesion | 5.23E-02 | TNC | TNC |

**Supplemental Table 4**. The BP terms associated with immune function were enriched from unique DEGs observed in the MH vs. M.

| Function | ID | Biological term | Number of gene | *P*-value |
| --- | --- | --- | --- | --- |
| Cytokine production | |  |  |  |
|  | GO:0001817 | Regulation of cytokine production | 10 | 0.009 |
|  | GO:0001816 | Cytokine production | 10 | 0.015 |
|  | GO:0007229 | Integrin-mediated signaling pathway | 4 | 0.025 |
|  | GO:0032715 | Negative regulation of interleukin-6 production | 3 | 0.030 |
|  | GO:0001819 | Positive regulation of cytokine production | 7 | 0.032 |
|  | GO:0001818 | Negative regulation of cytokine production | 5 | 0.040 |
|  | GO:0032720 | Negative regulation of tumor necrosis factor production | 3 | 0.042 |
|  | GO:0032675 | Regulation of interleukin-6 production | 4 | 0.042 |
|  | GO:1903556 | Negative regulation of tumor necrosis factor superfamily cytokine production | 3 | 0.044 |
|  | GO:0032635 | Interleukin-6 production | 4 | 0.046 |
| Immune response | |  |  |  |
|  | GO:0002252 | Immune effector process | 13 | 0.001 |
|  | GO:0006955 | Immune response | 16 | 0.001 |
|  | GO:0002682 | Regulation of immune system process | 16 | 0.005 |
|  | GO:0050776 | Regulation of immune response | 10 | 0.008 |
|  | GO:0002697 | Regulation of immune effector process | 8 | 0.012 |
|  | GO:0002683 | Negative regulation of immune system process | 7 | 0.025 |
|  | GO:0002263 | Cell activation involved in immune response | 5 | 0.034 |
|  | GO:0002253 | Activation of immune response | 6 | 0.035 |
|  | GO:0002684 | Positive regulation of immune system process | 10 | 0.035 |
|  | GO:0002460 | Adaptive immune response based on somatic recombination of immune receptors built from immunoglobulin superfamily domains | 5 | 0.038 |
| Immunocyte activation | |  |  |  |
|  | GO:0007159 | Leukocyte cell-cell adhesion | 9 | 0.008 |
|  | GO:0046649 | Lymphocyte activation | 10 | 0.012 |
|  | GO:0045321 | Leukocyte activation | 10 | 0.026 |
|  | GO:0002521 | Leukocyte differentiation | 8 | 0.033 |
|  | GO:0002366 | Leukocyte activation involved in immune response | 5 | 0.034 |
|  | GO:0042110 | T cell activation | 7 | 0.042 |
|  | GO:0070489 | T cell aggregation | 7 | 0.042 |
|  | GO:0071593 | Lymphocyte aggregation | 7 | 0.043 |
|  | GO:0070486 | Leukocyte aggregation | 7 | 0.047 |
| Toll-like receptor signaling pathway | | | |  |
|  | GO:0034154 | Toll-like receptor 7 signaling pathway | 2 | 0.046 |
|  | GO:0034121 | Regulation of toll-like receptor signaling pathway | 3 | 0.048 |
| Others |  |  |  |  |
|  | GO:0006952 | Defense response | 14 | 0.016 |

**Supplemental Table 5**. The BP terms associated with nutrient transport and metabolism were enriched from unique DEGs observed in the MC vs. M.

| Function | ID | Biological term | Number of genes | *P*-value |
| --- | --- | --- | --- | --- |
| Ion transport | |  |  |  |
|  | GO:0071804 | cellular potassium ion transport | 9 | <0.001 |
|  | GO:0071805 | potassium ion transmembrane transport | 9 | <0.001 |
|  | GO:0032413 | negative regulation of ion transmembrane transporter activity | 7 | <0.001 |
|  | GO:0010959 | regulation of metal ion transport | 18 | <0.001 |
|  | GO:0043269 | regulation of ion transport | 21 | <0.001 |
|  | GO:0043271 | negative regulation of ion transport | 9 | <0.001 |
|  | GO:0006811 | ion transport | 36 | <0.001 |
|  | GO:1904063 | negative regulation of cation transmembrane transport | 7 | <0.001 |
|  | GO:0030001 | metal ion transport | 25 | <0.001 |
|  | GO:0098660 | inorganic ion transmembrane transport | 17 | 0.001 |
|  | GO:0034766 | negative regulation of ion transmembrane transport | 7 | 0.001 |
|  | GO:0006812 | cation transport | 27 | 0.001 |
|  | GO:0006813 | potassium ion transport | 9 | 0.001 |
|  | GO:0098655 | cation transmembrane transport | 17 | 0.001 |
|  | GO:0098662 | inorganic cation transmembrane transport | 16 | 0.001 |
|  | GO:2001257 | regulation of cation channel activity | 7 | 0.002 |
|  | GO:0034220 | ion transmembrane transport | 18 | 0.003 |
|  | GO:0030004 | cellular monovalent inorganic cation homeostasis | 6 | 0.004 |
|  | GO:0055067 | monovalent inorganic cation homeostasis | 8 | 0.004 |
|  | GO:0006820 | anion transport | 12 | 0.005 |
|  | GO:0032412 | regulation of ion transmembrane transporter activity | 10 | 0.006 |
|  | GO:1904062 | regulation of cation transmembrane transport | 11 | 0.011 |
|  | GO:0015672 | monovalent inorganic cation transport | 12 | 0.013 |
|  | GO:0043270 | positive regulation of ion transport | 10 | 0.014 |
|  | GO:0015698 | inorganic anion transport | 5 | 0.015 |
|  | GO:1901016 | regulation of potassium ion transmembrane transporter activity | 5 | 0.015 |
|  | GO:0034765 | regulation of ion transmembrane transport | 12 | 0.021 |
|  | GO:0051924 | regulation of calcium ion transport | 9 | 0.032 |
|  | GO:0051453 | regulation of intracellular pH | 4 | 0.038 |
|  | GO:1901017 | negative regulation of potassium ion transmembrane transporter activity | 3 | 0.039 |
|  | GO:0030641 | regulation of cellular pH | 4 | 0.043 |
|  | GO:0006873 | cellular ion homeostasis | 16 | 0.044 |
|  | GO:1901379 | regulation of potassium ion transmembrane transport | 5 | 0.046 |
| Nitrogen transport and metabolism | | |  |  |
|  | GO:0071705 | nitrogen compound transport | 23 | <0.001 |
|  | GO:0042398 | cellular modified amino acid biosynthetic process | 5 | 0.002 |
|  | GO:0042886 | amide transport | 13 | 0.004 |
|  | GO:0015833 | peptide transport | 12 | 0.008 |
|  | GO:0071901 | negative regulation of protein serine/threonine kinase activity | 8 | 0.008 |
|  | GO:0015844 | monoamine transport | 5 | 0.024 |
|  | GO:0006563 | L-serine metabolic process | 3 | 0.031 |
|  | GO:2001056 | positive regulation of cysteine-type endopeptidase activity | 7 | 0.037 |
|  | GO:0006584 | catecholamine metabolic process | 4 | 0.038 |
|  | GO:0009712 | catechol-containing compound metabolic process | 4 | 0.038 |
|  | GO:0010950 | positive regulation of endopeptidase activity | 7 | 0.039 |
|  | GO:0055086 | nucleobase-containing small molecule metabolic process | 21 | 0.040 |
|  | GO:0072522 | purine-containing compound biosynthetic process | 10 | 0.041 |
|  | GO:0010952 | positive regulation of peptidase activity | 7 | 0.044 |
|  | GO:0052547 | regulation of peptidase activity | 10 | 0.046 |
|  | GO:0042423 | catecholamine biosynthetic process | 3 | 0.048 |
|  | GO:0009713 | catechol-containing compound biosynthetic process | 3 | 0.048 |
|  | GO:0009070 | serine family amino acid biosynthetic process | 3 | 0.048 |
| Lipid transport and metabolism | | |  |  |
|  | GO:0006633 | fatty acid biosynthetic process | 9 | 0.005 |
|  | GO:0006629 | lipid metabolic process | 37 | 0.010 |
|  | GO:0032370 | positive regulation of lipid transport | 5 | 0.019 |
|  | GO:0006631 | fatty acid metabolic process | 12 | 0.031 |
|  | GO:0006869 | lipid transport | 11 | 0.038 |
|  | GO:0032368 | regulation of lipid transport | 6 | 0.044 |
|  | GO:0060192 | negative regulation of lipase activity | 3 | 0.048 |
| Organic acid metabolism | | |  |  |
|  | GO:0016053 | organic acid biosynthetic process | 17 | <0.001 |
|  | GO:0046394 | carboxylic acid biosynthetic process | 16 | <0.001 |
|  | GO:0072330 | monocarboxylic acid biosynthetic process | 10 | 0.005 |
|  | GO:0019752 | carboxylic acid metabolic process | 27 | 0.006 |
|  | GO:0043436 | oxoacid metabolic process | 27 | 0.006 |
|  | GO:0006082 | organic acid metabolic process | 28 | 0.013 |
|  | GO:0032787 | monocarboxylic acid metabolic process | 16 | 0.044 |
| Phosphate transport and metabolism | | |  |  |
|  | GO:0045936 | negative regulation of phosphate metabolic process | 23 | 0.002 |
|  | GO:0010563 | negative regulation of phosphorus metabolic process | 23 | 0.002 |
|  | GO:0006796 | phosphate-containing compound metabolic process | 73 | 0.010 |
|  | GO:0006793 | phosphorus metabolic process | 73 | 0.010 |
|  | GO:0010519 | negative regulation of phospholipase activity | 3 | 0.012 |
|  | GO:0009165 | nucleotide biosynthetic process | 13 | 0.013 |
|  | GO:1901293 | nucleoside phosphate biosynthetic process | 13 | 0.017 |
|  | GO:0032780 | negative regulation of ATPase activity | 3 | 0.018 |
|  | GO:0006753 | nucleoside phosphate metabolic process | 21 | 0.020 |
|  | GO:0019220 | regulation of phosphate metabolic process | 48 | 0.020 |
|  | GO:0051174 | regulation of phosphorus metabolic process | 48 | 0.020 |
|  | GO:0043462 | regulation of ATPase activity | 4 | 0.029 |
|  | GO:0090407 | organophosphate biosynthetic process | 16 | 0.032 |
|  | GO:0009117 | nucleotide metabolic process | 19 | 0.048 |
| Substance transport | |  |  |  |
|  | GO:0032410 | negative regulation of transporter activity | 7 | 0.001 |
|  | GO:0051049 | regulation of transport | 55 | 0.001 |
|  | GO:0034763 | negative regulation of transmembrane transport | 7 | 0.001 |
|  | GO:0022898 | regulation of transmembrane transporter activity | 10 | 0.008 |
|  | GO:0032409 | regulation of transporter activity | 10 | 0.012 |
|  | GO:0051051 | negative regulation of transport | 18 | 0.015 |
|  | GO:0055085 | transmembrane transport | 21 | 0.023 |
|  | GO:0034762 | regulation of transmembrane transport | 12 | 0.029 |
|  | GO:0051348 | negative regulation of transferase activity | 10 | 0.035 |
|  | GO:0051050 | positive regulation of transport | 29 | 0.043 |
| Other substance transport and metabolism | | |  |  |
|  | GO:0044283 | small molecule biosynthetic process | 21 | <0.001 |
|  | GO:0050790 | regulation of catalytic activity | 48 | 0.001 |
|  | GO:0043086 | negative regulation of catalytic activity | 22 | 0.001 |
|  | GO:0015850 | organic hydroxy compound transport | 10 | 0.005 |
|  | GO:0018958 | phenol-containing compound metabolic process | 7 | 0.006 |
|  | GO:0051336 | regulation of hydrolase activity | 25 | 0.007 |
|  | GO:0051186 | cofactor metabolic process | 15 | 0.012 |
|  | GO:1901615 | organic hydroxy compound metabolic process | 14 | 0.012 |
|  | GO:0051346 | negative regulation of hydrolase activity | 12 | 0.020 |
|  | GO:0006821 | chloride transport | 4 | 0.025 |
|  | GO:1901617 | organic hydroxy compound biosynthetic process | 8 | 0.026 |
|  | GO:0006732 | coenzyme metabolic process | 12 | 0.032 |
|  | GO:0009108 | coenzyme biosynthetic process | 7 | 0.039 |
|  | GO:0046189 | phenol-containing compound biosynthetic process | 4 | 0.048 |
|  | GO:0030574 | collagen catabolic process | 3 | 0.048 |


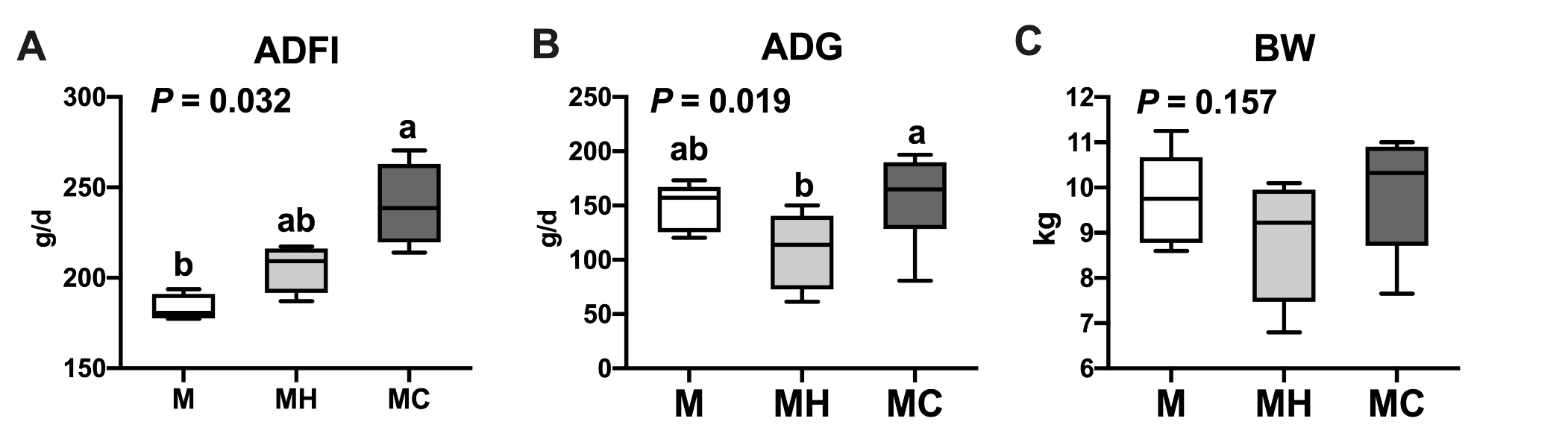
**Supplemental Fig. 1** Effect of alfalfa hay or concentrate starter on the ADFI (A), ADG (B), and BW (C) of pre-weaned lambs. Values are shown as means ± pooled SEM, *n* = 8. Mean values within a column with unlike superscript letters were significantly different (*P* < 0.05). The ADFI was measured based on dry matter. M, goat milk group; MH, goat milk plus alfalfa hay group; MC, goat milk plus concentrate diet group; ADFI, average daily feed intake; ADG, average daily gain; BW, body weight.


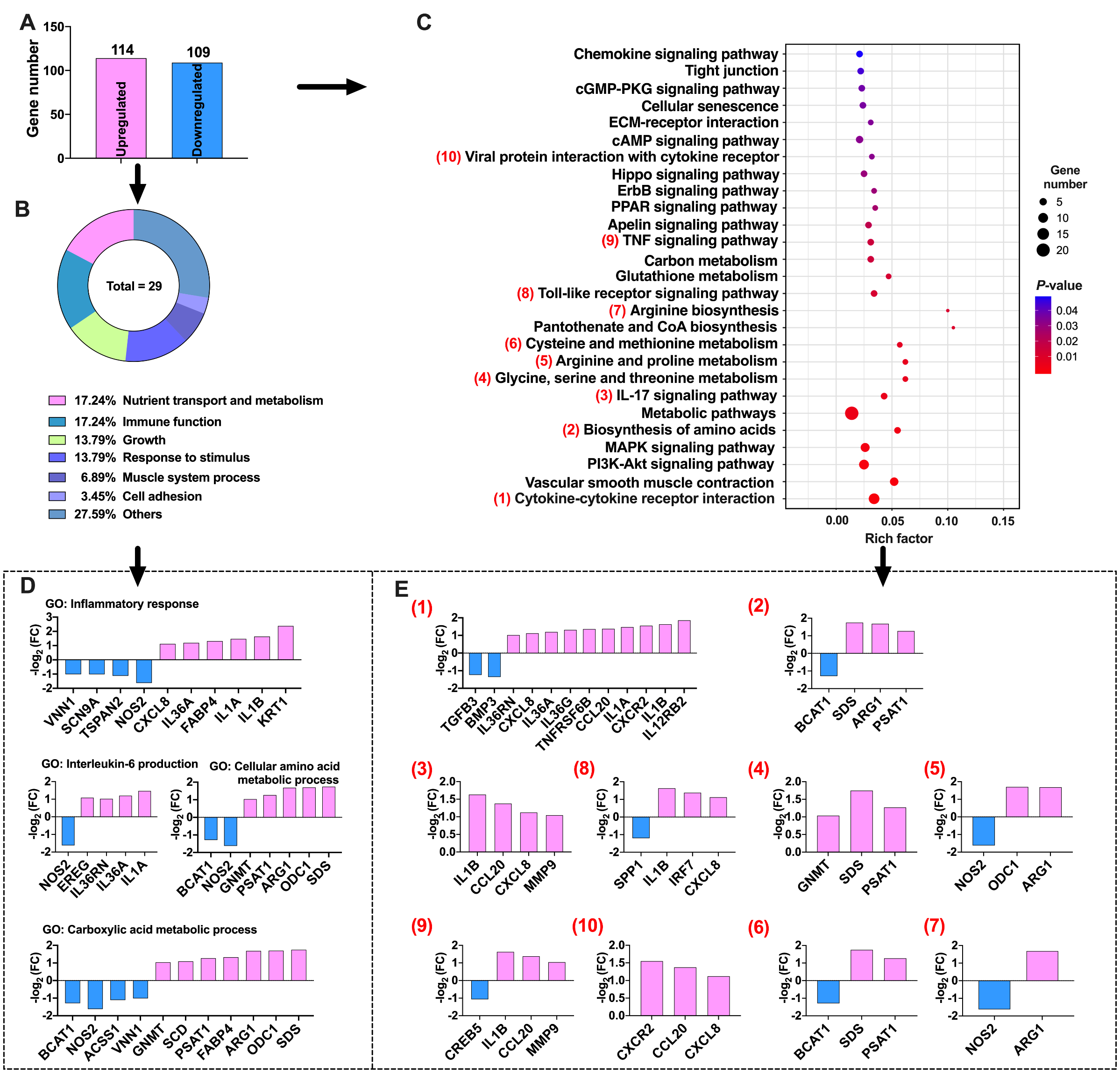


**Supplemental Fig. 2** The comparison of MC and MH in transcriptome analysis. A. The number of DEGs identified in MC vs MH. B. The significant biological process classification of DEGs. C. The significantly enriched KEGG pathways of DEGs. D. Genes expression in mainly biological terms of DEGs. E. Genes expression in mainly KEGG pathways of DEGs. An adjusted *P* < 0.05 and an absolute value of [log_2_ (fold change)] > 1 were set as the filter criteria for significant differential expression genes (*n* = 4 per group). The significance of identified KEGG pathways was determined by *P* < 0.05.
